# Developing the PIP-eco: An integrated genomic pipeline for identification and characterization of *Escherichia coli* pathotypes encompassing hybrid forms

**DOI:** 10.1016/j.csbj.2024.07.017

**Published:** 2024-07-20

**Authors:** Seyoung Ko, Huynh Minh Triet Nguyen, Woojung Lee, Donghyuk Kim

**Affiliations:** a School of Energy and Chemical Engineering, Ulsan National Institute of Science and Technology (UNIST), Ulsan 44919, Republic of Korea; b School of Life Sciences, Ulsan National Institute of Science and Technology (UNIST), Ulsan 44919, Republic of Korea; c Division of Food Microbiology, National Institute of Food and Drug Safety Evaluation, Ministry of Food and Drug Safety, Cheongju 28159, Republic of Korea

**Keywords:** *Escherichia coli*, Hybrid pathotype, Classification tool, Comprehensive analysis

## Abstract

Pathogenic *Escherichia coli* (*E. coli*) strains are distinguished by their diverse virulence factors, which contribute to a wide spectrum of diseases. These pathogens evolve through the horizontal transfer of virulence factors, resulting in the emergence of hybrid pathotypes with complex and heterogeneous characteristics. Recognizing their profound impact on public health, this study introduces the PIP-eco pipeline, a comprehensive analytical tool designed for the precise identification and characterization of *E. coli* pathotypes. This PIP-eco pipeline advances beyond traditional molecular techniques by facilitating detailed analysis of both single and hybrid pathotypes. It integrates targeted marker gene analysis, virulence factor-based phylogenetic analysis, and pathogenicity islands (PAIs) profiling to elucidate the genetic diversity of *E. coli* pathotypes and support their accurate classification. This integrative approach enables PIP-eco to uncover connections among various *E. coli* pathotypes, highlight shared virulence factors, and provide insights into their evolutionary trajectories. By utilizing experimentally validated marker genes, the pipeline ensures robust identification of pathotypes, particularly those of hybrid pathotypes. Additionally, PAI analysis offers comprehensive genetic investigations, revealing strain-specific variations and potential virulence mechanisms. As a result, the PIP-eco pipeline emerges as a useful tool for dissecting the evolutionary dynamics of *E. coli* and characterizing complex pathotypes, addressing the critical need for accurate detection and understanding of hybrid pathotypes.

## Graphical Abstract

**Figure:**
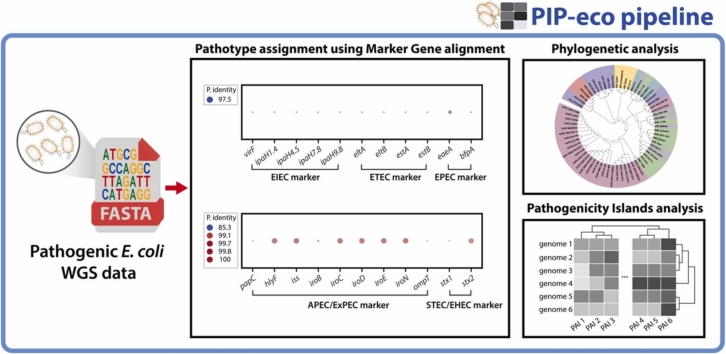

## 1 Importance

The rising prevalence of hybrid pathotypes in *Escherichia coli* underscores the need for advanced tools for accurate pathogen identification and characterization. The PIP-eco pipeline addresses this critical requirement by offering a comprehensive approach to analyze *E. coli* pathotypes using Whole Genome Sequencing (WGS) data. This pipeline integrates three essential analytical components: marker gene analysis, phylogenetic analysis, and pathogenicity islands (PAIs) profiling. By combining these methodologies, PIP-eco facilitates the precise identification of both single and hybrid pathotypes while providing in-depth insights into their genetic composition and virulence mechanisms. The capability of the pipeline to identify and characterize hybrid pathotypes is particularly significant, given the diverse characteristics and potential risks associated with the hybridization of *E. coli* pathotypes. This comprehensive approach provided by the PIP-eco pipeline not only elucidates pathogenic properties and evolutionary background but also establishes a new framework for identification and characterization of hybrid forms.

## 2 Introduction

*Escherichia coli*, a bacterium primarily implicated in both intestinal and extraintestinal diseases, has a substantial impact on human health [1], [2], [3], [4]. This bacterial species comprises multiple pathotypes, each defined by a unique combination of virulence factors (VFs) and pathogenic mechanisms [5]. These pathogenic *E. coli* strains are classified into distinct pathotypes, also known as pathovars, which exhibit comprehensive characteristics including specific VFs, host specificity, and clinical manifestations [6], [7], [8]. Consequently, the accurate identification of *E. coli* pathotypes is essential for understanding the epidemiology, diagnosis, and treatment of *E. coli* infections [9].

Traditionally, the classification of *E. coli* pathotypes has relied on phenotypic characteristics such as toxin production, attachment patterns to epithelial cells, and clinical symptoms [3], [10], [11], [12]. While these criteria have provided a foundational understanding of the diverse pathogenic mechanisms of *E. coli*, they are limited in specificity and sensitivity [13], [14], [15], [16]. This limitation has driven the development of molecular techniques aimed at more precise and targeted pathotype identification [17], [18]. The introduction of PCR-based methods, in particular, has enabled accurate and rapid pathotyping through the detection of specific virulence genes [19], [20], [21], [22]. Furthermore, advancements in technologies such as microarrays and film arrays have facilitated the simultaneous analysis of multiple genes, offering a comprehensive overview of the genetic composition within strains [23], [24], [25], [26], [27]. These advancements have improved the understanding of pathogenic mechanisms by identifying and comparing virulence gene profiles across multiple pathotypes [28].

Despite these advances, relying on a limited number of marker genes has drawbacks in accurately reflecting the complexity of pathotypes due to the sharing of genetic characteristics and various VFs among *E. coli* pathotypes [29]. Horizontal gene transfer (HGT) between different strains of *E. coli* and other bacteria leads to the sharing of virulence factors from different pathotypes [30]. Recent studies have highlighted the emergence of hybrid pathotypes that combine virulence factors from different pathotypes [31], [32]. These hybrid pathotypes arise through HGT events, such as the acquisition of PAIs or phage-related toxin genes [33], [34]. The 2011 outbreak in Germany caused by a hybrid pathotype underscores the importance of detecting and characterizing these emerging pathotypes [35], [36].

Given the significance and complexity of hybrid pathotypes, a comprehensive approach to pathotype analysis is essential. A key element in understanding both traditional and hybrid pathotypes is the characterization of PAIs. Generally, *E. coli* pathotypes are characterized by the presence and structure of their PAI-related genes, which reflect the specific virulence mechanisms of each pathotype [37], [38], [39]. For instance, the Locus of Enterocyte Effacement (LEE) PAI, associated with the effacement of microvilli on enterocytes and intimate attachment, is present in both EPEC (Enteropathogenic *E. coli*) and EHEC (Enterohemorrhagic *E. coli*) pathotypes [40]. Moreover, since some PAIs are strain-specific [41], [42], identifying specific PAIs can effectively discriminate *E. coli* pathotypes [43]. Therefore, it is crucial to consider the possibility of hybrid pathotypes in *E. coli* pathotype analysis and to develop methods that accurately identify them, with a particular focus on characterizing specific PAIs. The advancement of Next-Generation Sequencing (NGS) technologies has revolutionized bacterial genomics, with Whole-Genome Sequencing (WGS) enabling a comprehensive understanding of pathogenicity by providing complete genomic information of pathogens [44], [45], [46]. Analyses utilizing WGS data are particularly effective in detecting and characterizing novel pathotypes, including hybrid pathotypes. However, the application of WGS data for *E. coli* pathotype identification has been limited due to the absence of integrated analysis tools. While existing relevant databases provide valuable information on *E. coli* pathotypes and support downstream analysis, they do not offer a systemic framework for identifying and characterizing PAIs within the context of *E. coli* pathotype analysis.

To address this gap, this study introduces the PIP-eco pipeline, developed specifically for *E. coli* pathotype analysis using WGS data. The PIP-eco pipeline delivers a comprehensive characterization of *E. coli* pathotypes by integrating bioinformatic analysis, alignment with data from previously established marker genes, and the characterization of VFs and PAIs. This multifaceted approach offers insights into pathotypes based on the evolutionary background and genetic composition of *E. coli* strains through the comparison and analysis of genomic data with phylogenetic and molecular markers. Furthermore, by providing detailed PAI profiles of *E. coli* strains, the pipeline aids in the identification of hybrid pathotypes and contributes to a foundational knowledge base essential for understanding their pathogenic mechanisms.

In summary, the PIP-eco pipeline serves as an effective tool for the identification and analysis of pathogenic *E. coli* with hybrid pathogenicity from multiple perspectives, thereby offering valuable insights into pathogenic mechanisms.

## 3 Materials and methods

### 3.1 Reference bacterial dataset collection and serotyping

Data on reference *Escherichia coli* were collected from various scientific sources. A comprehensive and iterative search of scientific literature was performed using PubMed and Google Scholar. The search utilized the following keywords related to various *E. coli* pathotypes, including “EAEC”, “DAEC”, “EPEC”, “EIEC”, “ETEC”, “EHEC”, “STEC”, “AIEC”, “UPEC”, “NMEC”, “APEC”, “ExPEC”, “InPEC” and “*E. coli* pathotype” and so on. Papers explicitly mentioning the pathotype name were screened, focusing on those where the pathotype of the corresponding reference strain was experimentally proven. From these screened papers, only strains with provided accession numbers were selected for further analysis, as these numbers were essential for accessing the genomic data. In the pathotype selection process, we focused on enabling detailed analysis based on complete genomic information. Therefore, only pathotypes with at least one strain having a complete genome were included in the study. Pathotypes that did not meet this criterion were excluded from the research. Eleven pathotypes met this criterion, and 10 strains were selected for each pathotype. In total, WGS data for 110 reference strains were downloaded from RefSeq database from NCBI to ensure reliability.

### 3.2 Data collection for genetic markers and pathogenicity islands

Data on marker genes for pathotype identification were collected through a comprehensive literature review. We searched the scientific literature through Google Scholar using the keyword "marker gene" for each pathotype. Genes closely associated with the pathogenesis mechanisms of each pathotype were collected, prioritizing those reported to have high sensitivity and specificity as markers. The specificity of these marker genes was evaluated by performing local alignment against collected reference genomes, leading to the final selection of the most specific marker genes for each pathotype. Additionally, for pathotypes with known main VFs, we verified the presence or absence of these factors in our constructed reference genome dataset for each pathotype. Nucleotide sequence information for the selected marker genes was obtained from the NCBI nucleotide database. While there were some databases related to PAIs, we primarily utilized PAIDB (v.2.0) [47], which provided species-specific PAI information. PAIDB was a web-based database that provided Genbank accession numbers for species-specific identified PAIs. Therefore, we searched for PAIs whose organism was "*Escherichia coli*" and crawled based on the provided accession number to obtain sequence data for PAI-associated genes. *E. coli*-specific PAIs were chosen to ensure high relevance to the organism [48]. The Entrez retriever available at NCBI was used to crawl the data. Finally, the collected PAI-associated gene sequence data were subjected to a curation procedure to identify duplicate sequences and integrate their annotations. A total of 2158 PAI-associated gene sequences comprising 97 PAIs were obtained, which was comprehensively utilized for PAI analysis.

### 3.3 Construction of analytic process for the PIP-eco pipeline

The PIP-eco pipeline integrated marker gene analysis, VF-based phylogenetic tree construction, and PAI analysis for *E. coli* pathotype identification and characterization. In marker gene analysis, pathotype-specific marker genes selected through literature review were used to identify the pathotype of the input genome. Local alignment of marker genes against the input genome was performed using USEARCH software [49]. Hits with nucleotide sequence identity of 80 % or higher and coverage of 50 % or more were considered valid. Each valid hit indicated the pathotype specified by the corresponding marker gene. By combining these hits, we determined whether the input genome represented a single pathotype or a hybrid pathotype. For VF-based phylogenetic tree construction, VFs in the input genome were first identified. Local alignment was performed on the input genome based on VFs confirmed in reference genomes of 11 pathotypes. Hits with nucleotide sequence identity of 80 % or higher and coverage of 90 % or more were considered valid VFs. All VF genes found in the genomes were used for phylogenetic tree construction. The tree construction process began with genome annotation using Prokka software (v1.11) [50]. The Prokka-annotated genomes were then used as input for pan-genome and phylogenetic analysis. This analysis was performed using the Panaroo software package (v.1.3.4) [51]. The initial clustering parameters of Panaroo were set to 60 % sequence identity threshold and 90 % length difference cutoff, with the clean-mode flag executed under the “moderate” algorithm. The VF based phylogenetic tree was constructed from the gene presence/absence matrix generated by Panaroo. This matrix underwent sequence alignment using the MAFFT software (v7.310) [52], followed by the application of the neighbor-joining method using MUSCLE software (v.3.8.31) [53] to generate the phylogenetic tree. The PAI analysis of the input genome was conducted using a dataset of PAI-related genes selected from *E. coli*. Local alignment was performed between the input genome and this dataset, with hits having amino acid sequence identity of 80 % or higher and coverage of 90 % or more considered valid. The PIP-eco pipeline provided positional information for each valid PAI-related gene hit. Based on this information, the continuity of PAI-related genes was evaluated, and gene grouping was performed. This process identified potential PAI boundaries. Additionally, the GC content of the input genome as host was compared with that of the detected PAI-related gene groups. This comparison assessed the possibility that the gene group was introduced from external sources through gene transfer. Significant difference in GC content suggested a high likelihood that the region was a mobile PAI.

During the initial construction of the PIP-eco pipeline, pan-genome analysis based on VFs detected in reference genomes was performed to understand the evolutionary context of pathogenic *E. coli*. Pan-genome analysis identified core (> 95 % prevalence), accessory (15 - 95 %), and unique (< 15 %) gene pools. A bootstrapping function was implemented to perform 100 random samplings to estimate the variability of core and pan-genome sizes. This process was used for pipeline development and validation and was not included in the user version. In addition, the phylogenetic tree was visualized using the interactive Tree of Life (iTOL) [54] v6.

### 3.4 Performance assessment of the PIP-eco pipeline for pathotype assignment

To evaluate the pathotype assignment performance of the PIP-eco pipeline, a dataset was constructed using independent WGS data samples. For the collection of WGS data, a keyword search for “pathotype pathogen” was conducted within the NCBI pathogen detection database. The WGS data pool was then created by filtering the organism group to “*E. coli* and *Shigella*” and excluding WGS data without accession numbers. To ensure a dataset that included a diverse range of *E. coli* species, a random selection process was implemented. Using a random number generation mechanism, 400 *E. coli* genomes were selected from the WGS data pool. To enhance the statistical reliability of the constructed dataset, a 10-fold stratified cross-validation approach was utilized. This process was repeated ten times, thereby establishing the dataset for performance testing. These ten datasets, each comprising 400 genomes, were subjected to the PIP-eco pipeline for evaluation across various metrics, including virulence factor detection, pathotype assignment, rates of unassigned genomes, and hybrid pathotype detection.

### 3.5 Validation test of PIP-eco pipeline using strains with hybrid pathotype

The ability of the PIP-eco pipeline to analyze hybrid pathotypes was validated using a set of strains with hybrid pathotypes. These strains were selected based on a comprehensive literature review. The selection criteria included strains whose hybrid pathotypes established through molecular techniques and for which accession numbers were available. Genomic data for the selected strains were retrieved from the NCBI RefSeq database using the provided accession numbers. The PIP-eco pipeline was then applied to these strains. Through the marker gene analysis, the hybrid pathotype character of each input strain was confirmed. Subsequently, these results were compared with the pathotypes reported in the literature to assess the accuracy of the pipeline in identifying hybrid strains. The genetic relationships of the hybrid test strains with known pathotypes were visualized using the VF-based phylogenetic tree of the pipeline. In this process, the pipeline utilized either the "panaroo-integrate" function (for analyzing a single genome) or the "panaroo-merged" function (for analyzing multiple genomes) after the Prokka annotation procedure. Phylogenetic associations between hybrid strains and established pathotypes were determined through this analysis. PAI analysis was performed on the input strains using the pipeline, and all detected PAI-related genes were collected for further analysis. The identified PAI-related genes were categorized into groups including T2SS-related genes, LEE PAI-related T3SS genes, ETT2 PAI-related T3SS genes, ExPEC virulence mechanism-related genes, and environmental adaptation-related genes.

## 4 Results

### 4.1 Development of pipeline for discriminating pathotypes in hybrid form

**Fig. 1:**
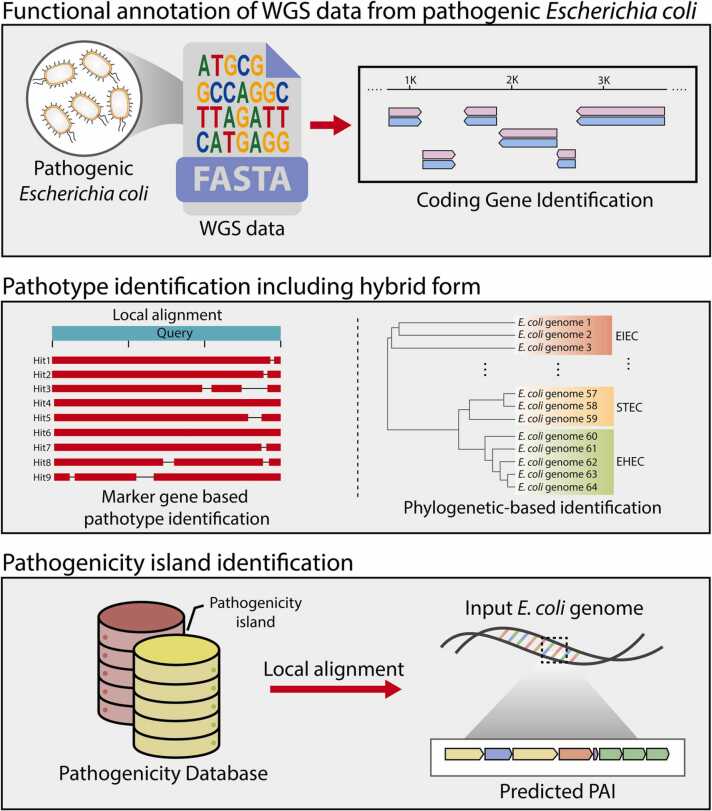
Overview of the PIP-eco pipeline workflow**.** The PIP-eco pipeline is a comprehensive framework designed for the classification and characterization of *E. coli* pathotypes, including hybrid forms. It consists of three key processes: functional annotation, pathotype identification, and pathogenicity island analysis. This structured approach not only facilitates the accurate classification of *E. coli* pathotypes but also deepens the understanding of potential hybridization events through the meticulous identification of pathogenicity islands.

The PIP-eco pipeline identifies and characterizes *E. coli* pathotypes using a three-step procedure: marker gene analysis, phylogenetic tree-based analysis, and PAI characterization (Fig. 1). In the first step, local alignment of identified marker gene sequences is performed to determine pathotypes. Marker genes specific to each pathotype, selected through a comprehensive literature review (Table S1), are compared with the input genome. This process confirms the pathotype of the input genome and detects potential hybrid pathotypes within *E. coli*, providing crucial information for subsequent analyses.

The second step of the PIP-eco pipeline differentiates *E. coli* pathotypes using a dataset of 110 reference genomes, representing 11 different pathotypes (Table S2). In this step, VF genes in the input genome are identified and compared with VFs from reference strains to construct a VF-based phylogenetic tree. This provides an essential foundation for an in-depth understanding of the multiple complexities of the *E. coli* bacterium and facilitates differentiation between pathotypes. This approach reveals extensive genomic diversity among pathogenic *E. coli* strains and provides a framework for determining which reference strains possess similar pathogenicity factors.

The final step involves identifying and analyzing PAIs in the input genome using known *E. coli* PAI information. Understanding that genes located on mobile PAIs can drive hybridization between different pathogens, the detailed PAI information provided by the pipeline serves as a basis for pathogen characterization. The analyzed PAIs offer significant insights into the pathogenic mechanisms of *E. coli*, extending beyond mere genetic markers and demonstrating the ability of the pipeline to elucidate the role of PAIs in hybridization events. Through this integrated approach, the PIP-eco pipeline provides a comprehensive method for analyzing and understanding the characteristics of multiple *E. coli* pathotypes. The analysis results from the PIP-eco pipeline include three main outputs (Fig. S1): (i) local alignment of genetic markers; (ii) VF-based phylogenetic tree analysis results, (iii) PAI information. These outputs enable users to interpret the characteristics of the analyzed genome and its relationship with reference genomes. Furthermore, for the analysis of large datasets, a Python script is available in a freely accessible repository. This script facilitates the identification of hybridized pathotypes from pathogen genomic data and the acquisition of unique PAI information.

Existing tools for *E. coli* pathotype identification using WGS data are limited. To evaluate the effectiveness of the PIP-eco pipeline, an independent analysis was conducted. A test dataset was constructed by randomly selecting 400 *E. coli* genomes from the NCBI pathogen database, and this process was iterated ten times to ensure statistical consistency. The analysis detected a total of 127,110 hits, from which 14,467 virulence genes were identified based on protein sequence identity above 80 % and 90 % coverage (Fig. S2A). Upon application of the pipeline, pathotypes were assigned to an average of 284 genomes, while 116 genomes were found to have no assigned pathotypes (Fig. S2B). The frequency of pathotypes was observed in the order of EHEC, ExPEC/AIEC group (including NMEC, UPEC, APEC and AIEC pathotypes), STEC, EIEC, EPEC, EAEC, DAEC, and ETEC. This detection pattern remained consistent across all iterations. Additionally, hybrid pathotypes were identified in an average of 13.4 genomes (Fig. S2C). Analysis of hybrid pathotypes revealed that the ExPEC/AIEC group was the most frequently observed, forming hybrid events with all pathotypes except EIEC. Notably, hybrid events between ExPEC/AIEC and STEC were the most frequent (Fig. S2D), aligning with the previous research that highlights their prevalence and significance [55], [56]. Consequently, the PIP-eco pipeline demonstrated stable performance across multiple *E. coli* genomes, with an assignment rate of 70.9 % (standard deviation: 2.94) for single pathotypes and 3.4 % (standard deviation: 1.32) for hybrid pathotypes.

### 4.2 Comprehensive analysis of *E. coli* strains with multiple pathotypes

The genetic markers used in the PIP-eco pipeline were selected to effectively discriminate among multiple pathotypes (Fig. S3). A total of 55 gene markers were chosen, focusing on genes directly associated with major disease processes (Table S1). Local alignment analysis of these selected marker genes was performed against reference genomes representing 11 pathotypes. This analysis revealed that InPEC (EAEC, DAEC, EPEC, EIEC, EHEC, STEC) could be specifically identified by their respective markers. For ExPEC (NMEC, UPEC, APEC), no individual pathotype-specific markers were identified; however, shared virulence factors were observed among them. Notably, for AIEC, previous studies have confirmed the absence of established specific markers. This finding was consistent with the marker gene analysis results of the PIP-eco pipeline, suggesting that many virulence factors are shared between AIEC and ExPEC. Based on this observation, the selected marker gene set was designed to identify ExPEC and AIEC as a combined group.

**Fig. 2:**
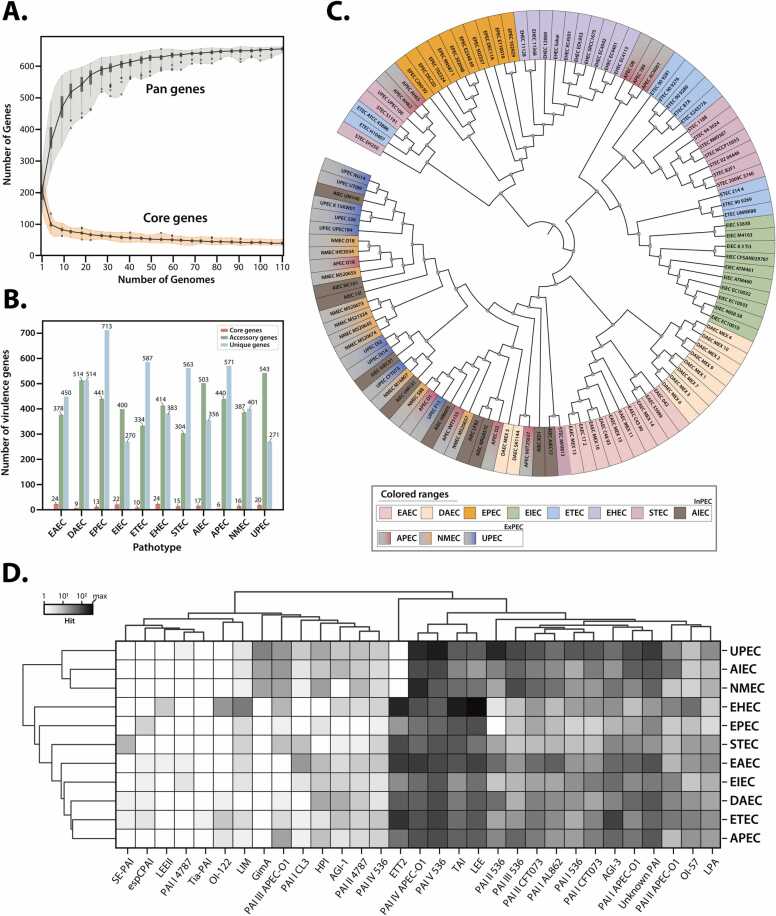
Genomic analysis outcomes for 110 *E. coli* strains across eleven pathotypes using the PIP-eco pipeline. (A) Pan-genome and core-genome trends: this part illustrates the evolution of pan-genome and core-genome among reference *E. coli* strains as determined by pan-genome analysis, revealing a closed pan-genome indicative of a comprehensive gene pool. The pan-genome encompasses the entire gene set, whereas the core genome includes genes present in all analyzed strains. (B) Distribution of core, accessory, and unique genes across different *E. coli* pathotypes. The bar graph displays the gene composition for each pathotype, with core (red), accessory (green), and unique (blue) genes. (C) Phylogenetic connections: a phylogenetic tree showcasing the relationships of VFs detected in 110 *E. coli* strains, with individual pathotypes distinguished by color coding. (D) Pathogenicity island distribution: a heatmap displaying the presence and spread of identified PAIs across each pathotype, utilizing colors to represent the log10-transformed counts of hits for each PAI.

To comprehensively evaluate the genetic diversity and relationships among multiple *E. coli* pathotypes, a pan-genome analysis was performed based on VFs identified in 110 reference genomes representing each pathotype (Fig. 2A). This analysis identified a total pan-genome size of 662 VF genes with 81 core genes (12.2 % with >95 % prevalence), 254 accessory genes (38.4 % with 15–95 % prevalence), and 327 unique genes (49.4 % with <15 % prevalence) [57], [58], [59]. The constructed VF-based phylogenetic tree (Fig. 2C) revealed distinct clustering patterns among the pathotypes. Within the InPEC group, distinct clusters were formed by EAEC, EIEC, EPEC, and EHEC. Meanwhile, DAEC, ETEC, STEC, and AIEC demonstrated genetic proximity to several other pathotypes, reflecting their genetic diversity and environmental adaptability [60]. In the ExPEC group, APEC, NMEC, and UPEC were closely clustered, with AIEC showing genetic proximity to the ExPEC pathotype. These results highlight the extensive genomic diversity among the multiple pathogenic *E. coli* strains.

A key feature of the PIP-eco pipeline is its ability to elucidate the pathogenicity patterns of *E. coli* pathotypes through additional PAI analysis. The PAI landscape of each pathotype was identified through local alignment with available PAI-associated gene data (Fig. 2D, Table S3), revealing 276 pathotype-specific PAI-related genes within 23 PAIs (Fig. S4). The PAI analysis indicated varying numbers of PAI-associated genes across pathotypes, with EHEC (94), EPEC (49), and STEC (27) showing particularly high numbers within the InPEC group. LEE PAI-related genes were abundantly found in EHEC and EPEC, including T3SS-related genes, intimin, and various effector proteins, reflecting their ability to form A/E lesions. Additionally, Efa1-LifA-Tox protein and Ent protein genes were found in EHEC, suggesting roles in immune evasion and additional toxin production, respectively. Enterotoxin (*esp*) and cytotoxin genes were identified in EPEC, demonstrating its toxin-producing capability. In STEC, subtilase cytotoxin subunit A and B, as well as putative adhesin/hemagglutinin/hemolysin genes, were identified, although Shiga toxin-related genes were not directly detected. The presence of SubAB toxin and the absence of LEE PAI-encoded T3SS suggested the potential of STEC for cytotoxic effects and less intimate adhesion compared to EHEC. Relatively fewer PAI-related genes were identified in DAEC, EAEC, EIEC, and ETEC. In EAEC, fimbrial protein and P pilus assembly protein PapJ were found, reflecting the aggregative adherence mechanism. PapE protein was primarily detected in DAEC, indicating its diffuse adherence capability. In EIEC, while genes directly related to the main pathogenic mechanism were not detected, the *cad* operon transcriptional activator was identified, suggesting an ability for environmental adaptation. Interestingly, ETEC exhibited genes primarily related to mobility elements, such as transposases, rather than specific VFs, suggesting high genetic plasticity among the analyzed InPEC pathotypes. Within the ExPEC group, UPEC exhibited the highest number of PAI-related genes, including those involved in adhesion and colonization (*Sfa* genes, *Pap* gene family), toxin production (*Hly* gene family), and immune evasion through encapsulation (*Kps* gene family). These findings accurately reflect the pathogenic mechanisms of UPEC specialized for urinary tract infections. In contrast, very few PAI-associated genes were identified in NMEC and APEC. Notably, a uropathogenic-specific protein was identified in AIEC, showing genetic similarity with UPEC and suggesting the potential for extra-intestinal infection. This finding aligns with the earlier observation of shared virulence factors between AIEC and ExPEC. In addition, environmental adaptation or mobility-related factors were identified across all 11 analyzed pathotypes.

The PIP-eco pipeline employs a multi-faceted analysis approach that combines marker gene identification, pan-genome analysis, and PAI profiling, thereby providing a comprehensive view of *E. coli* pathotype diversity. Marker gene analysis effectively discriminated between different pathotypes, while pan-genome analysis based on VFs identified in reference strains revealed the extent of genetic variation among strains. Additionally, PAI analysis elucidated the specific virulence mechanisms of each pathotype. Together, these analyses not only highlight the genetic diversity and complex relationships among *E. coli* pathotypes but also offer valuable insights into their evolutionary trajectories and adaptive strategies.

### 4.3 Validation of hybrid pathotype analysis in the PIP-eco pipeline

To validate the capability of the PIP-eco pipeline for hybrid pathotype analysis, 17 *E. coli* strains known to possess hybrid pathogenic properties were selected and analyzed based on a literature review (Table S4). Marker gene analysis revealed that 16 out of 17 strains exhibited pathotypes consistent with previous studies (Table S5). However, the EH3155 strain, previously identified as STEC/ExPEC, displayed characteristics of EHEC/ExPEC upon marker gene analysis.

**Fig. 3:**
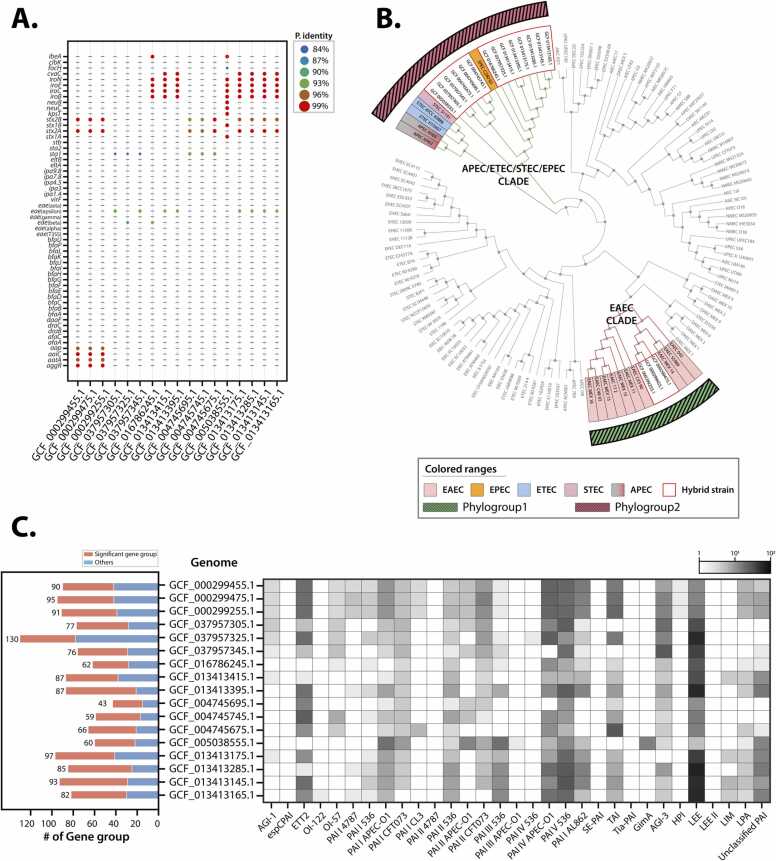
Hybrid pathotype identification and PAI distribution analysis using the PIP-eco pipeline **(**A) Marker gene local alignment outcomes: showcasing the alignment results for marker genes within 17 *E. coli* strains identified as having hybrid pathotypes. (B) Phylogenetic analysis of 17 *E. coli* strains with hybrid pathotypes: The phylogenetic tree is color-coded to indicate different pathotypes. Two main clusters are highlighted: Phylogroup 1 (EAEC clade) and Phylogroup 2 (APEC/ETEC/STEC/EPEC clade). (C) Distribution of PAI-related genes and genome comparison: Bar plot (left) showing the total number of PAIs found in each genome. Gene groups with a GC content difference of more than 1.5 % compared to the GC content of the host genome are labeled as “Significant gene group”, indicating externally acquired gene groups. Heatmap (right) displaying the presence (dark gray) or absence (white) of gene groups across different *E. coli* genomes. Rows represent individual hybrid strains, and columns represent specific PAI characteristics. The heatmap illustrates the distribution, using colors to denote the log10-transformed frequency of each PAI.

Phylogenetic analysis demonstrated that the 17 strains formed distinct clusters, classified into two phylogroups (Fig. 3B): phylogroup 1 (EAEC clade) and phylogroup 2 (APEC/ETEC/STEC/EPEC clade). Specifically, three genomes (2011 C-3493, 2009EL-2071, 2009EL2050) were classified into phylogroup 1, while the remaining strains were assigned to phylogroup 2.

In the PAI analysis using the PIP-eco pipeline, consecutively arranged PAI-related genes were considered as a single putative PAI. Gene groups with GC content significantly different from the overall GC content of the host genome were classified as significant groups, presumed to be externally acquired. Analysis of the resulting PAI profiles showed that three strains belonging to phylogroup 1 had highly similar PAI environments. These strains had nearly identical numbers of putative PAIs (90 to 95), PAI-associated genes (306 to 313), and significant groups (48 to 53). In contrast, the 14 strains in phylogroup 2 displayed considerable variability in their PAI profiles, with putative PAI numbers ranging from 42 to 130, PAI-associated gene counts from 110 to 363, and significant group numbers from 28 to 66.

Detailed analysis of PAI-related genes was conducted to understand the genetic characteristics and potential pathogenicity of each pathotype group. This analysis revealed a diverse distribution of virulence and adaptation-related gene clusters (Table S6). EAEC/STEC hybrid pathotype strains (2011 C-3493, 2009EL-2071, 2009EL-2050) showed highly similar PAI profiles, characterized by T2SS and ETT2 PAI-related genes, along with environmental adaptation genes. In EPEC/ETEC hybrid pathotype strains (MFDS1009724, MFDS1017696, MFDS1017708), LEE PAI-related T3SS gene clusters were consistently observed. Notably, the MFDS1017696 strain was found to possess additional ETT2 PAI-related genes and iron acquisition system genes, distinguishing it from the other two strains. EPEC/ExPEC hybrid pathotype strains (BA1250, EH2219, EH3322) were identified as having LEE PAI and ETT2 PAI-related genes, as well as ExPEC virulence-related genes. Additional ExPEC-related genes and immune evasion genes were detected in EH3319 and EH3322 strains, differentiating them from the BA1250 strain. In STEC/ETEC hybrid pathotype strains (SE572, SE573, SE574), ETT2-PAI-related genes and environmental adaptation genes were predominantly observed. The SE574 strain was found to possess additional adaptation genes, setting it apart from the other two strains. In the STEC/ExPEC hybrid pathotype strain FWSEC0527, the presence of ExPEC-related genes and environmental adaptation genes was found, but LEE PAI-related genes characteristic of EHEC strains were not identified.

Interestingly, the EH3155 strain, previously annotated as STEC/ExPEC, was reclassified as EHEC/ExPEC through the marker gene analysis of the PIP-eco pipeline. This reclassification was further supported by the PAI analysis results. The PAI profile of EH3155 was found to be highly similar to those of known EHEC/ExPEC strains (EH3310, EH3338, EH3320), with common features including LEE PAI and ETT2 PAI-related genes, ExPEC-related genes, and environmental adaptation genes being identified. The results of the PAI analysis reveal strain-specific differences within the same hybrid pathotypes group, indicating diverse gene acquisition and loss events during the evolution of pathogenic *E. coli*. Notably, a correlation exists between the PAI profiles and the genetic relationships observed. Strains that clustered together in the phylogenetic tree exhibited similar PAI profiles, further corroborating their evolutionary connections. The distribution patterns of T3SS, ExPEC, and adaptation-related genes underscore the pathogenic potential and environmental adaptability of each strain.

In conclusion, the PIP-eco pipeline successfully identified and characterized the complex features of *E. coli* pathogens through the integration of marker gene analysis, phylogenetic analysis, and PAI analysis. This comprehensive approach facilitated the confirmation of existing pathotypes, the reclassification of misclassified strains, and the elucidation of differences between strains within the same pathotypes.

## 5 Discussion

The identification of pathogenic *Escherichia coli* strains is crucial for public health and disease management. There has been a notable increase in epidemiological significance of *E. coli* with hybrid pathotypes in both intestinal and extraintestinal diseases [32], [61], [62]. The emergence of hybrid strains can be attributed to the rapid dissemination of VFs and active gene exchange between pathogens [31], [63], [64]. This rise in these hybrid strains necessitates further research to accurately assess their disease burden. Although conventional molecular techniques have demonstrated high sensitivity and specificity in pathogen identification, the increasing prevalence of hybrid strains presents challenges for accurate identification and characterization. In response, improved molecular techniques, such as film array, have been developed to enable simultaneous detection of multiple VFs and facilitate the identification of some hybrid strains. However, from a long-term perspective, the utilization of WGS data is recognized to offer significant advantages, providing comprehensive genetic information and enabling more extensive analysis compared to traditional molecular techniques. Despite these advantages, there is currently a lack of tools to comprehensively analyze and characterize *E. coli* pathogens using WGS data.

To address this need, the PIP-eco pipeline was developed. This pipeline integrates marker gene, phylogenetic, and PAI analyses to provide a comprehensive method for understanding the characteristics of *E. coli* pathotypes. Analysis of randomly selected *E. coli* WGS datasets has demonstrated the performance of this pipeline in identifying both single and hybrid pathotypes, with 3.4 % of analyzed data identified as hybrid pathotypes. This prevalence is slightly higher than the 1.7 % to 2.1 % reported in recent studies [65], [66], [67], [68]. Interestingly, our analysis revealed that STEC/ExPEC hybrids were the most frequently detected hybrid pathotype. This finding is particularly noteworthy in light of recent studies that have described the emergence of such hybrid strains as a recent phenomenon [32], [55]. The high detection rate of these newly emerging hybrid strains in our study not only validates the sensitivity of the PIP-eco pipeline but also suggests that these hybrid pathotypes may be more prevalent than previously recognized. This underscores the importance of continued surveillance and highlights the potential epidemiological significance of these emerging hybrid strains in public health.

A key feature of the PIP-eco pipeline is its use of marker genes for pathotype identification. These genes, selected through a comprehensive literature review, accurately reflect major pathogenic mechanisms, thus enabling precise pathotype identification [61], [69], [70], [71], [72], [73], [74], [75], [76], [77], [78], [79], [80], [81], [82], [83], [84], [85], [86], [87]. The pipeline further enhances characterization through phylogenetic and PAI analyses, which may facilitate the discovery of novel pathotype-specific virulence factors. A reference strain dataset for eleven pathotypes has been constructed, supporting VF-based phylogenetic analysis. Although the dataset includes a limited number of genomes, it provides clear evolutionary background information by incorporating at least one complete genome for each pathotype. Furthermore, the PAI analysis within the pipeline has yielded valuable insights into specific pathotypes. For instance, ETEC strains exhibit close genetic relationships with other pathotypes and contain multiple transposases and IS elements, indicating a high potential for hybridization and environmental adaptation. The effectiveness of the PIP-eco pipeline in analyzing hybrid pathotypes has been validated through the examination of 17 known hybrid strains, achieving high accuracy in confirming previously reported pathotypes and suggesting more precise classification for certain strains. Notably, the analysis of highly virulent hybrid strains, such as EAEC/STEC, revealed that these strains possess an EAEC backbone with enhanced adaptation capabilities. The PAI profile analysis has uncovered differences between strains within the same hybrid pathotype, indicating diverse evolutionary processes in pathogenic *E. coli*. The observation that PAI-related genes in EAEC/STEC strains show low variability and high conservation aligns with previous findings of limited diversity in these strains. The distribution patterns of T3SS, ExPEC, and adaptation-related genes in analyzed hybrid pathotype strains reflect the pathogenic potential and environmental adaptability of each strain, carrying significant clinical and public health implications.

The integrated approach of the PIP-eco pipeline has facilitated not only the confirmation of previous pathotypes but also the reclassification of misclassified strains and the identification of intra-pathotype differences. These capabilities enhance the accuracy of *E. coli* pathogen classification, deepen our understanding of pathogenic mechanisms, and aid in the early detection and response to emerging pathogenic *E. coli* strains. Nevertheless, the integration of additional data sources is imperative, as the current dataset does not adequately represent genes involved in major pathogenic mechanisms of some pathogens, including plasmid-mediated or phage-mediated virulence factors. It is important to note that the focus of the PIP-eco pipeline on *E. coli* PAI may limit its applicability to PAIs across different bacterial species, as highlighted in prior reviews [48]. Further characterization of ExPEC and AIEC pathotypes is crucial due to their shared virulence factors and close genetic relationships. The absence of specific markers to clearly distinguish these pathotypes highlights the necessity for a more comprehensive analytical approach. Consequently, an expanded number of reference genomes is critical for achieving more accurate differentiation and characterization of these pathotypes.

In conclusion, the PIP-eco pipeline has proven to be a valuable tool for multifaceted analysis of the complex genetic characteristics of *E. coli* hybrid pathotypes. This pipeline is expected to provide critical insights into the evolutionary and adaptation mechanisms of *E. coli* pathogens, thereby informing the development of effective public health strategies.

## Funding

This work was supported by the 10.13039/501100003725National Research Foundation of Korea (NRF) funded by the Ministry of Science and ICT (MSIT [2022M3A9I5018934, 2021M3A9I4024840]. In addition, this research was supported by the Ministry of Food and Drug Safety (MFDS), South Korea [20161MFDS030].

## Author contribution

Conceptualization: S.K., W.L., and D.K. Methodology: S.K., H.M.T.N. Software development: S.K and H.M.T.N. Validation: S.K and W.L. Processed computational analysis: S.K. and H.M.T.N. Writing— original draft preparation: S.K., and D.K. Writing— review and editing: D.K.

## CRediT authorship contribution statement

**Seyoung Ko:** Writing – original draft, Visualization, Validation, Software, Methodology, Investigation, Formal analysis, Data curation, Conceptualization. **Donghyuk Kim:** Writing – review & editing, Writing – original draft, Supervision, Conceptualization. **Woojung Lee:** Validation, Conceptualization. **Huynh Minh Triet Nguyen:** Software, Methodology.

## Declaration of Competing Interest

None declared.

## Data Availability

The source code for the PIP-eco pipeline is freely available under an open-source license at https://github.com/SBML-Kimlab/PIP-eco.
