# Supplementary material for: Developing the PIP-eco: An integrated genomic pipeline for identification and characterization of Escherichia coli pathotypes encompassing hybrid forms
Source: Comput Struct Biotechnol J. 2024 Jul 20;23:3040–9. doi: 10.1016/j.csbj.2024.07.017 (PMC11340603; doi:10.1016/j.csbj.2024.07.017)
Supplement: Supplementary file 1 — Supplementary material [file mmc1.docx]

# Supplementary materials


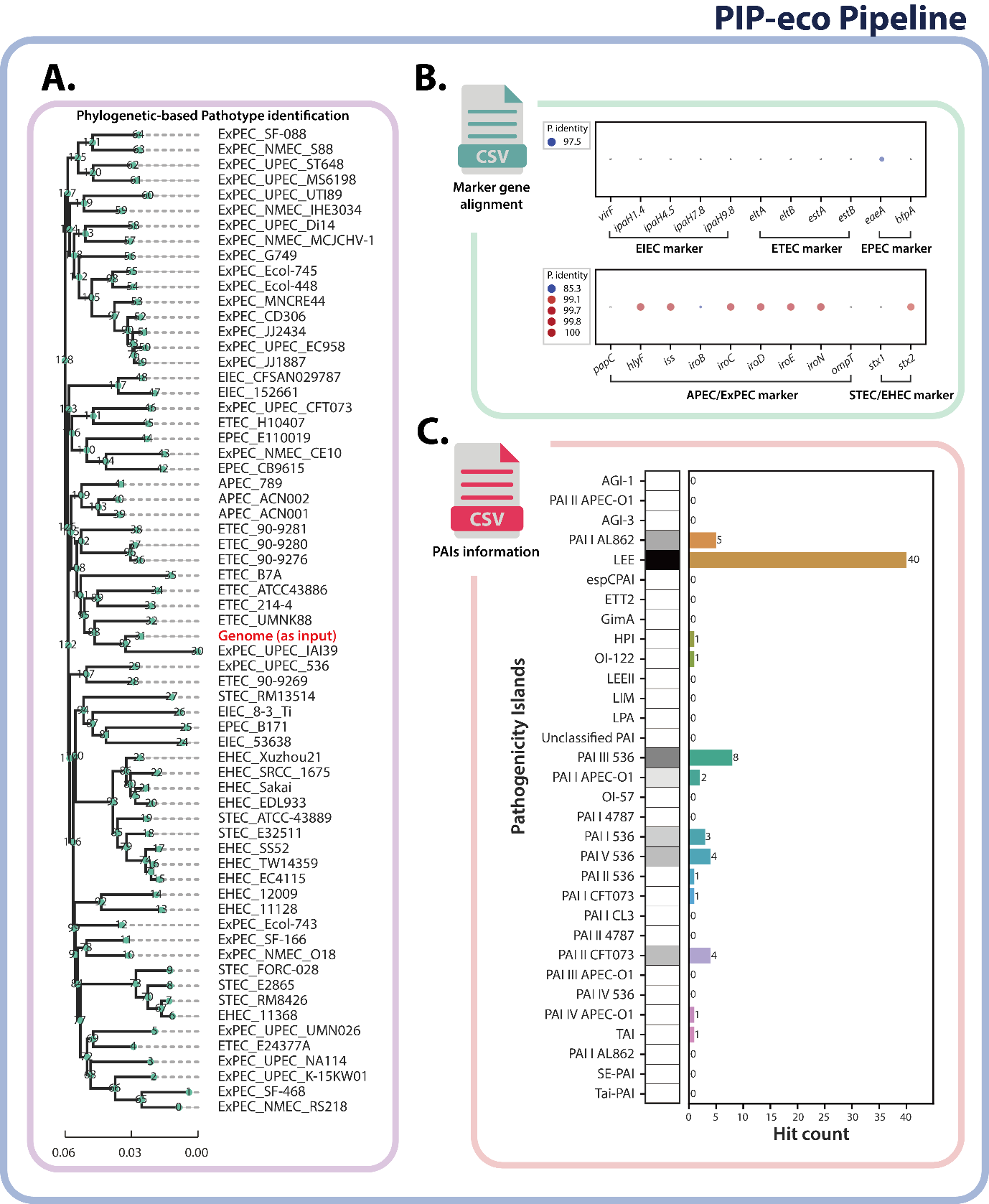


**Supplementary Figure S1. The output overview output of PIP-eco pipeline** The PIP-eco pipeline processes bacterial genome data input by users and conducts a three-stage analysis to yield classification results. (A) The first process integrates the input genome data into a constructed pan genome-based phylogenetic tree, resulting in the classification of pathotypes. The result of this classification is provided as a visualized phylogenetic tree showing the location of the input genome within the diverse genetic landscape. (B) In the second step for classification, the input genome is locally aligned to the marker genes. The alignment result is not only available in a tab-delimited CSV format for detailed examination, but also visualized as scatter plots to facilitate an intuitive understanding of pathotype identification. (C) Finally, the PIP-eco pipeline deliver the result from the pathogenicity islands (PAIs) analysis sin the third step. This analysis performs a local alignment based on the presence of unique PAIs associated with various pathotypes. The alignment result is provided in both a tab-delimited CSV format and visualized as bar charts and heatmaps, providing a comprehensive view of the pathogenic potential in the genome.


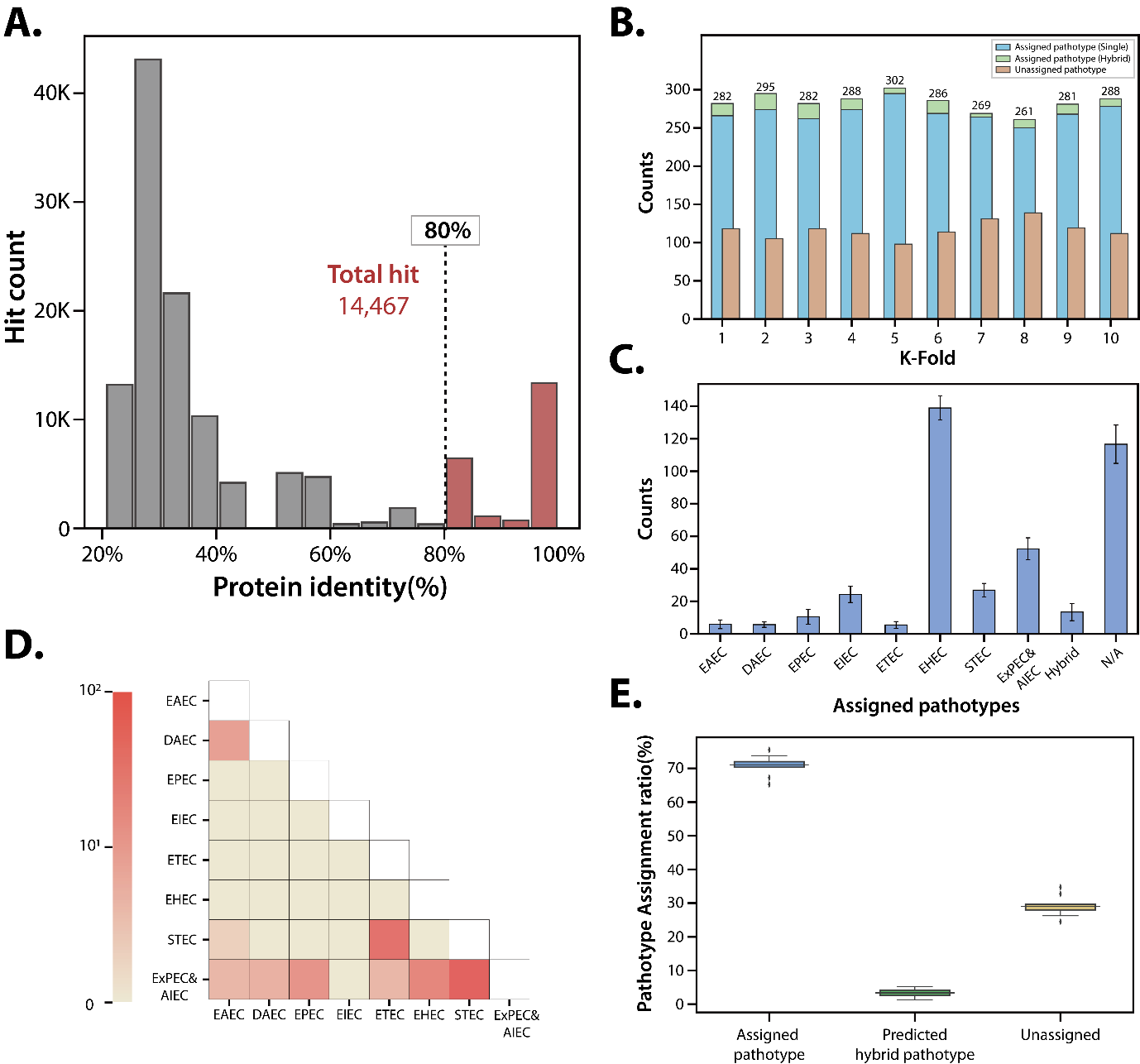


**Supplementary Figure S2. Assessment results of PIP-eco pipeline for pathotype assignment in E. coli strains** (A) Analysis of pathogenic *E. coli* strains corresponding to a total of 4,000 WGS confirmed that 14,467 virulence genes with protein similarity of 80% and 90% of coverage or more were detected. (B) The PIP-eco pipeline underwent a 10-fold stratified validation on the constructed dataset to assess the robustness of pathotype assignment. Strains with pathotype assignments, strains with hybrid pathotype assignments, and strains without pathotype assignments are color-coded (Blue: strains with pathotype assignments, green: strains with hybrid pathotype assignments, orange: strains without pathotype assignments). (C) The pathotype assignment within dataset, as determined by the PIP-eco pipeline, is displayed. The number of strains belonging to each category was counted: single, hybrid, and not assigned (N/A) (single pathotype: EAEC, DAEC, EPEC, EIEC, ETEC, EHEC, ExPEC&AIEC, hybrid pathotype, not assigned is displayed as "N/A"). (D) The PIP-eco pipeline facilitated the identification of hybrid events between different pathotypes within the dataset. Presented in a diagonal heatmap, the log10 transformed data shows the frequency of co-occurrence of different pathotypes. (E) The consistency of the PIP-eco pipeline in assigning pathotypes within a diverse set of E. coli strains is illustrated. The graph reflects the distribution of ratios for correctly assigned pathotypes, predicted hybrid pathotypes, and unassigned cases.


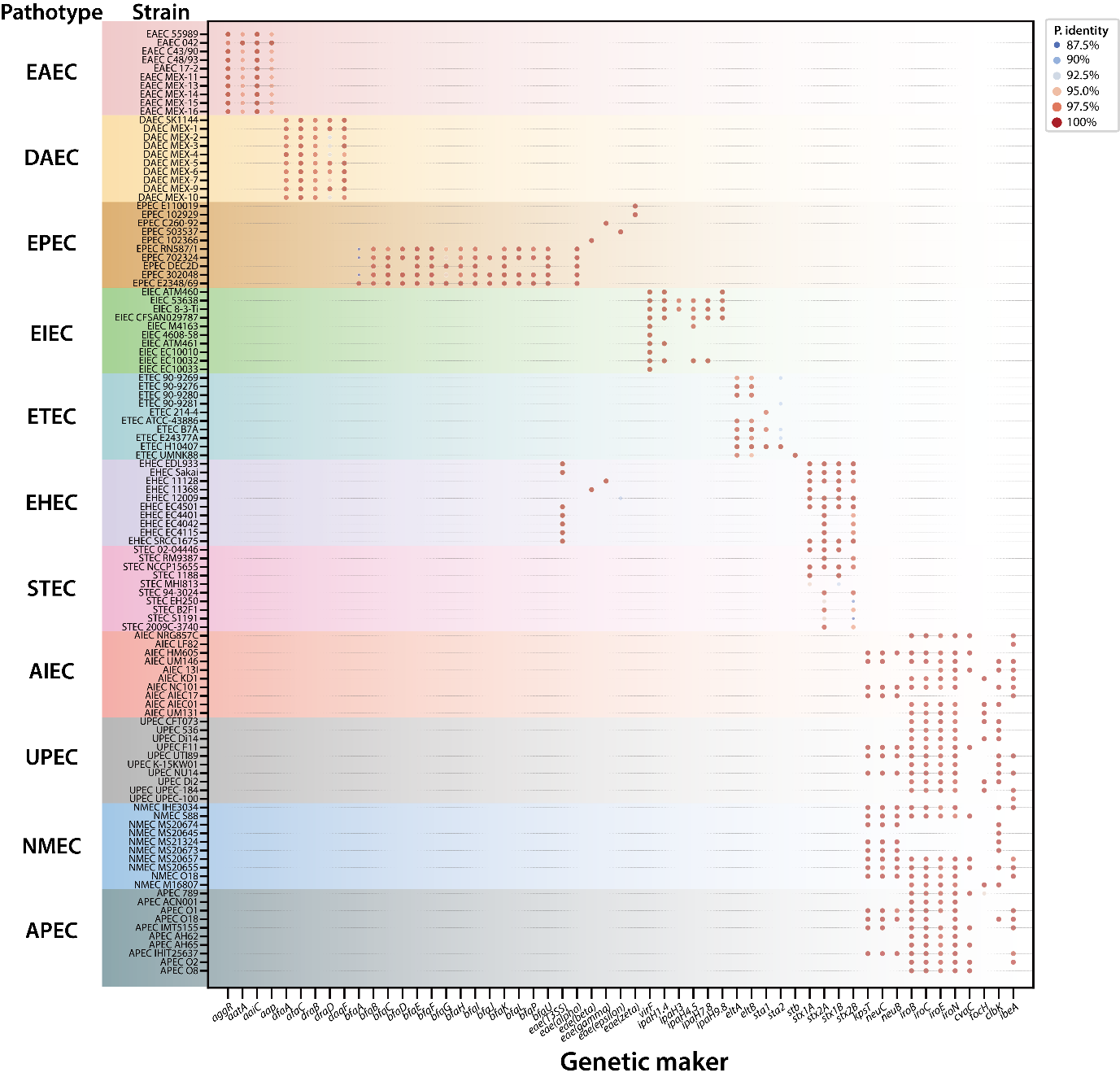


**Supplementary Figure S3. The results of local alignment using selected genetic markers across 64 reference *E. coli* strains with eleven pathotypes** The PIP-eco pipeline utilizes 55 selected genetic markers to identify pathotypes. The performance of these genetic marker to discriminate among pathotypes is demonstrated across 110 reference strains representing eleven pathotypes. The hits for each genetic marker within the strains are displayed in color and size-coded based on protein similarity.

**
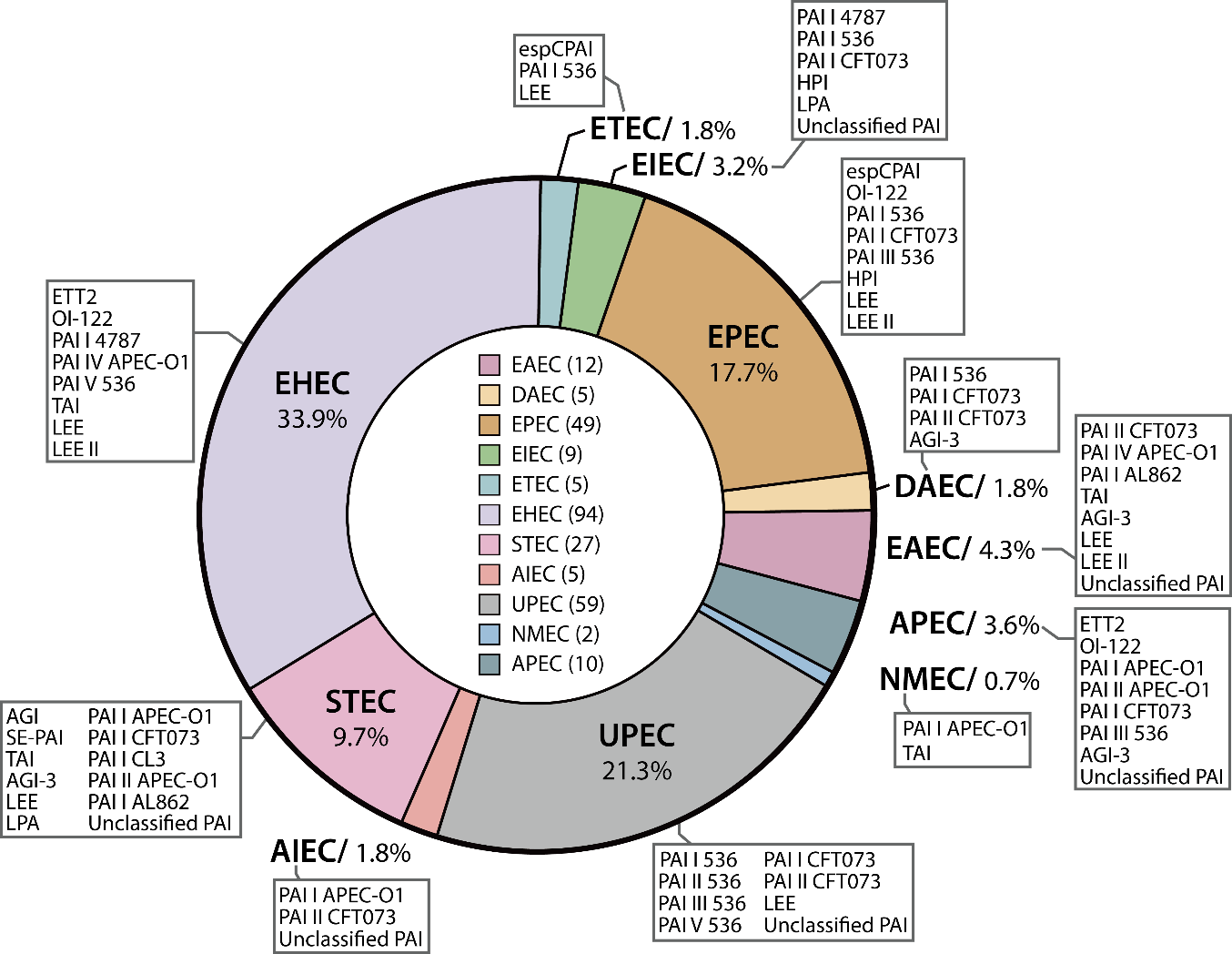
**

**Supplementary Figure S4. The distribution of unique pathogenicity islands across various pathotypes** It shows a comprehensive overview of the distribution of unique pathogenicity islands (PAIs) identified within seven *E. coli* pathotypes, as characterized by the PIP-eco pipeline. Each pathotype is color-coded, and the unique PAIs found within these pathotypes are delineated in boxes.
